# Supplementary material for: Knowledge, perceptions and attitude of Egyptian physicians towards biobanking issues
Source: PLoS One. 2021 Mar 26;16(3):e0248401. doi: 10.1371/journal.pone.0248401 (PMC7996976; doi:10.1371/journal.pone.0248401)
Supplement: S1 Table — (DOCX) [file pone.0248401.s001.docx]

**S1 Table: Background characteristics of the respondents (n = 223)**

| **Variables** | | **Number** | **Percent** |
| --- | --- | --- | --- |
| **Demographic variables:** | | | |
| **Age in years (Mean ± SD)*** | | 36 ± 8.4 | |
| **Gender** | |  |  |
|  | **Male** | 59 | 26.5 |
|  | **Female** | 164 | 73.5 |
| **Residence** | |  |  |
|  | **Cairo** | 105 | 47.1 |
|  | **Alexandria** | 84 | 37.7 |
|  | **Assiut** | 34 | 15.2 |
|  | |  | |
| **Years of Experience (Mean ± SD)*** | | 11 ± 7.9 | |
| **Affiliation** | |  |  |
|  | **University Staff** | 167 | 74.9 |
|  | **Not a University Staff (Master/ MD candidate)** | 56 | 25.1 |
| **Specialty** | |  |  |
|  | **Clinical pathology** | 67 | 30.0 |
|  | **Histopathology** | 23 | 10.3 |
|  | **Public Health& Community Medicine** | 45 | 20.2 |
|  | **Surgery** | 14 | 6.3 |
|  | **Anesthesia** | 8 | 3.6 |
|  | **Internal medicine** | 32 | 14.3 |
|  | **Basic Sciences** | 34 | 15.2 |
| **Biobanking-related variables:** | |  |  |
| **Type of current/last research** | |  |  |
|  | **Master** | 105 | 47.1 |
|  | **Doctoral** | 48 | 21.5 |
|  | **Post-doctoral** | 70 | 31.4 |
| **I am working/planning to work on blood samples for my current/future research** | | | |
|  | **Yes** | 96 | 43 |
|  | **No** | 97 | 35.4 |
|  | **Not sure** | 48 | 21.5 |
| **I am working/planning to work on tissue samples for my current/future research** | | | |
|  | **Yes** | 85 | 38.1 |
|  | **No** | 78 | 35 |
|  | **Not sure** | 60 | 26.9 |
| **I am working/planning to work on saliva/urine samples for my current/future research** | | | |
|  | **Yes** | 56 | 25.1 |
|  | **No** | 100 | 44.8 |
|  | **Not sure** | 67 | 30.1 |
| **I have attended a lecture/course/conference about Biobanking before** | | | |
|  | **Yes** | 46 | 20.6 |
|  | **No** | 177 | 76.4 |

*Quantitative variables are presented as Mean ± SD
