# Supplementary material for: Knowledge, perceptions and attitude of Egyptian physicians towards biobanking issues
Source: PLoS One. 2021 Mar 26;16(3):e0248401. doi: 10.1371/journal.pone.0248401 (PMC7996976; doi:10.1371/journal.pone.0248401)
Supplement: S2 Table — (DOCX) [file pone.0248401.s002.docx]

**S2 Table: The relation between knowledge about biobanking and demographic characteristics of the respondents**

| **Tested Variables** | **Category** | **Heard about "Biobanking" before** | | | | **P value*** |
| --- | --- | --- | --- | --- | --- | --- |
|  |  | **Yes (n=142)** | | **No (n=81)** | |  |
|  |  | **N** | **%** | **N** | **%** |  |
| **Age** | <**30** | 21 | 47.7 | 23 | 52.3 | 0.066 |
|  | **30≤40** | 91 | 68.4 | 42 | 31.6 |  |
|  | **40≤50** | 16 | 59.3 | 11 | 40.7 |  |
|  | **>50** | 14 | 73.7 | 5 | 26.3 |  |
| **Gender** | **Male** | 45 | 76.3 | 14 | 23.7 | **0.019**** |
|  | **Female** | 97 | 59.1 | 67 | 40.9 |  |
| **Residence** | **Cairo** | 67 | 63.8 | 38 | 36.2 | 0.179 |
|  | **Alexandria** | 49 | 58.3 | 35 | 41.7 |  |
|  | **Assiut** | 26 | 76.5 | 8 | 23.5 |  |
| **Years of Experience** | <**10** | 73 | 61.3 | 46 | 38.7 | 0.720 |
|  | **10≤20** | 56 | 67.5 | 27 | 32.5 |  |
|  | **20≤30** | 10 | 66.7 | 5 | 33.3 |  |
|  | **≥30** | 3 | 50.0 | 3 | 50.0 |  |
| **Affiliation** | **University Staff** | 109 | 65.3 | 58 | 34.7 | 0.393 |
|  | **Not a University Staff (Master/ MD candidate)** | 33 | 58.9 | 23 | 41.1 |  |
| **Specialty** | **Clinical pathology** | 48 | 71.6 | 19 | 28.4 | 0.262 |
|  | **Histopathology** | 11 | 47.8 | 12 | 52.2 |  |
|  | **Public Health& Community Medicine** | 25 | 55.6 | 20 | 44.4 |  |
|  | **Surgery** | 9 | 64.3 | 5 | 35.7 |  |
|  | **Anesthesia** | 7 | 87.5 | 1 | 12.5 |  |
|  | **Internal medicine** | 21 | 65.6 | 11 | 34.4 |  |
|  | **Basic Sciences** | 21 | 61.8 | 13 | 38.2 |  |

*Based on the results of chi square test

**Significant variables at P value **≤** 0.05
