# Supplementary material for: Knowledge, perceptions and attitude of Egyptian physicians towards biobanking issues
Source: PLoS One. 2021 Mar 26;16(3):e0248401. doi: 10.1371/journal.pone.0248401 (PMC7996976; doi:10.1371/journal.pone.0248401)
Supplement: S3 Table — (DOCX) [file pone.0248401.s003.docx]

**S3 Table: The relation between knowledge about biobanking and biobanking related variables of the respondents**

| **Tested Variables** | **Category** | **Heard about "Biobanking" before** | | | | **P value*** |
| --- | --- | --- | --- | --- | --- | --- |
|  |  | **Yes (n=142)** | | **No (n=81)** | |  |
|  |  | **N** | **%** | **N** | **%** |  |
| **Type of current/last research** | **Master** | 62 | 59.0 | 43 | 41.0 | 0.083 |
|  | **Doctoral** | 28 | 58.3 | 20 | 41.7 |  |
|  | **Post-doctoral** | 52 | 74.3 | 18 | 25.7 |  |
| **I am working/planning to work on blood samples for my current/future research** | **Yes** | 64 | 66.7 | 32 | 33.3 | 0.611 |
|  | **No** | 47 | 59.5 | 32 | 40.5 |  |
|  | **Not sure** | 31 | 64.6 | 17 | 35.4 |  |
| **I am working/planning to work on tissue samples for my current/future research** | **Yes** | 58 | 68.2 | 27 | 31.8 | 0.532 |
|  | **No** | 47 | 60.3 | 31 | 39.7 |  |
|  | **Not sure** | 37 | 61.7 | 23 | 38.3 |  |
| **I am working/planning to work on saliva/urine samples for my current/future research** | **Yes** | 36 | 64.3 | 20 | 35.7 | 0.884 |
|  | **No** | 62 | 62.0 | 38 | 38.0 |  |
|  | **Not sure** | 44 | 65.7 | 23 | 34.3 |  |
| **I have attended a lecture/course/conference about Biobanking before** | **Yes** | 46 | 100 | 0 | 0.0 | **<0.001**** |
|  | **No** | 100 | 56.5 | 77 | 43.5 |  |

*Based on the results of chi square test

**Significant variables at P value **≤** 0.05
