# Supplementary material for: Knowledge, perceptions and attitude of Egyptian physicians towards biobanking issues
Source: PLoS One. 2021 Mar 26;16(3):e0248401. doi: 10.1371/journal.pone.0248401 (PMC7996976; doi:10.1371/journal.pone.0248401)
Supplement: S1 File — (DOCX) [file pone.0248401.s004.docx]

**Background information provided for participants**

A biobank is a type of biorepository that stores biological samples (usually human) for use in research. Since 1990 biobanks have become an important resource in medical research, supporting many types of contemporary research like genomics and personalized medicine.

Biobanks give researchers access to data representing a large number of people. Samples in biobanks and the data derived from those samples can often be used by multiple researchers for cross purpose research studies. For example, many diseases are associated with single-nucleotide polymorphisms, and performing genome-wide association studies using large collections of samples which represent tens or hundreds of thousands of individuals can help to identify disease biomarkers. Many researchers struggled to acquire sufficient samples prior to the advent of biobanks.

Although biobanks may be confused with cornea banks and stem cell banks, the purpose of each of them is different. Biobanks store samples like blood, tissue, urine and saliva only for future research on diagnostics and therapeutics in different diseases.

Biobanks have provoked questions on privacy, research ethics and medical ethics. While viewpoints on what constitutes appropriate biobank ethics diverge, consensus has been reached that operating biobanks without establishing carefully considered governing principles and policies could be detrimental to communities that participate in biobank programs.

In 2009, The TIME magazine considered Biobanks among "10 Ideas Changing the World Right Now" because ""Biobanks will transform the way we see disease developing."

For more information about biobank and bioanking please check these links below

http://content.time.com/time/specials/packages/article/0,28804,1884779_1884782_1884766,00.html

http://www.bbc.com/news/health-17553931

https://newsnetwork.mayoclinic.org/discussion/five-facts-about-mayo-clinic-biobank/

The following video is a TEDx talk by a physician at National Cancer Institute, Cairo University about biobanking

https://youtu.be/ePHc0IC2Kjw

In Egypt, several biobanks have been established recently. These biobanks can provide support for medical research here. This survey aims at assessment of knowledge and attitude of researchers towards this new concept. So, your opinion really matters. By completing this questionnaire, you agree to participate with us in this study

# **Survey about Egyptian Physicians' knowledge and attitude towards Biobanking Issues**

1. **Background characteristics of the participants**

| **Demographic variables** | | | |
| --- | --- | --- | --- |
| 1. Age in years |  |  |  |
| 1. Gender | 1. Male | 1. Female |  |
| 1. Residence | 1. Cairo | 1. Alexandria | 1. Assiut |
| 1. Years of Experience |  |  |  |
| 1. Affiliation | 1. University Staff | 1. Not University Staff (Master or MD candidate) |  |
| 1. Specialty | 1. Clinical pathology | 1. Histopathology | 1. Public Health& Community Medicine |
|  | 1. Surgery | 1. Anesthesia | 1. Internal medicine |
|  | 1. Basic Sciences |  |  |
| **Biobanking-related variables** |  |  |  |
| 1. Type of current/last research | 1. Master | 1. Doctorate | 1. Post-doctoral |
| 1. I am working/planning to work on tissue samples for my current/future research | 1. Yes | 1. No | 1. Not sure |
| 1. I am working/planning to work on saliva/urine samples for my current/future research | 1. Yes | 1. No | 1. Not sure |
| 1. I have attended a lecture/course/conference about Biobanking before | 1. Yes | 1. No |  |

1. **Basic knowledge about Biobanking among the respondents**

| **Question** | **Yes** | **No** |
| --- | --- | --- |
| 1. I have heard the term "Biobanking" before |  |  |
| 1. There is a biobank in my institute |  |  |
| 1. There are several biobanks in Egypt |  |  |
| 1. There is a law that governs biobank work in Egypt |  |  |

1. **General attitudes of participants towards biobanking**

| **Question** | **Strongly Agree** | **Agree** | **Unsure** | **Disagree** | **Strongly Disagree** |
| --- | --- | --- | --- | --- | --- |
| 1. I think that the presence of biobanks is important for the development of new treatments |  |  |  |  |  |
| 1. I think that the presence of biobanks is important for the development of new methods of diagnosis |  |  |  |  |  |
| 1. I think biobanks can make a difference in biomedical research in general |  |  |  |  |  |
| 1. I think biobanks can make a difference in aspects related to quality of samples and data provided for research |  |  |  |  |  |
| 1. In the future, I will be interested in applying to get samples for my research from the biobank |  |  |  |  |  |
| 1. I would be willing to help create a biobank in my institute |  |  |  |  |  |
| 1. I will donate samples myself and will ask my relatives to donate samples to the biobank |  |  |  |  |  |
| 1. If there is a biobank in my institution, I will inform my patients about it |  |  |  |  |  |
| 1. I would like more information about biobanks to be more readily available |  |  |  |  |  |
| 1. If there is a course/ lecture/conference about Biobanking, I will be interested in attending |  |  |  |  |  |
| 1. Donating samples for research is in line with religious beliefs |  |  |  |  |  |

1. **Opinions of participants about issues related to privacy, sharing and access issues**

| **Question** | **Strongly Agree** | **Agree** | **Unsure** | **Disagree** | **Strongly Disagree** |
| --- | --- | --- | --- | --- | --- |
| 1. Biobanks can share samples and data with international research organizations |  |  |  |  |  |
| 1. Biobanks can share samples and data with commercial and pharmaceutical companies |  |  |  |  |  |
| 1. The biobank may provide any medical information to insurance companies |  |  |  |  |  |
| 1. The biobank may provide any medical information to treating physician |  |  |  |  |  |
| 1. The biobank can provide any medical information to government |  |  |  |  |  |
| 1. The biobank can provide confidential medical information to legal authorities if asked |  |  |  |  |  |
| 1. I will not donate my samples to a biobank because my identity could be known through my DNA |  |  |  |  |  |

1. **Opinions of participants about issues related to governance of biobanks**

| **Question** | **Strongly Agree** | **Agree** | **Unsure** | **Disagree** | **Strongly Disagree** |
| --- | --- | --- | --- | --- | --- |
| 1. A participant who donates blood for scientific research on genes and environment remains in control over his/her blood |  |  |  |  |  |
| 1. Biobanks owns the stored samples |  |  |  |  |  |
| 1. Biobanks are just in custody of the samples, but don’t own them. |  |  |  |  |  |
| 1. Biobanks may charge user fees for samples that are distributed to researchers. |  |  |  |  |  |
| 1. A transparent policy for distribution of samples to researchers should exist. |  |  |  |  |  |

1. **Opinions of participants about the informed consent and participant’s rights**

| **Question** | **Strongly Agree** | **Agree** | **Unsure** | **Disagree** | **Strongly Disagree** |
| --- | --- | --- | --- | --- | --- |
| 1. A broad consent that doesn’t include every future research is more suitable for biobank work. |  |  |  |  |  |
| 1. Participant information and samples stored in the biobank should be protected and securely stored. |  |  |  |  |  |
| 1. Sample donors should be compensated by some means for their samples |  |  |  |  |  |
| 1. Sample donors should be informed in detail how their samples will be used |  |  |  |  |  |
| 1. Sample donors should be informed if their samples will be transferred abroad |  |  |  |  |  |
| 1. Sample donors should be informed about results resulting from research on their samples |  |  |  |  |  |
