# Supplementary material for: Knowledge, perceptions and attitude of Egyptian physicians towards biobanking issues
Source: PLoS One. 2021 Mar 26;16(3):e0248401. doi: 10.1371/journal.pone.0248401 (PMC7996976; doi:10.1371/journal.pone.0248401)
Supplement: Attachment — Submitted filename: PONE-D-20-34081_reviewer_CCS.pdf [file pone.0248401.s005.pdf]

PLOS ONE

Knowledge, Perceptions and Attitude of Egyptian Physicians towards Biobanking Issues

--Manuscript Draft--

**Manuscript Number:**	PONE-D-20-34081
**Article Type:**	Research Article
**Full Title:**	Knowledge, Perceptions and Attitude of Egyptian Physicians towards Biobanking Issues
**Short Title:**	Egyptian Physicians' Opinions about Biobanking
**Corresponding Author:**	Ahmed Samir Abdelhafiz, PhD<br>National Cancer Institute Cairo University<br>Cairo, EGYPT
**Keywords:**	Biobanking; Egypt; Physicians; Perception; Attitude
**Abstract:**	Objectives: Collection and storage of biospecimens and data for biobanking raise many ethical concerns. Stakeholders' opinions about these ethical issues are important since they can help in the development of ethical guidelines to govern biobanking activities. Physicians are among the important stakeholders since they contact potential participants and could be biobank users. The goal of this study is to evaluate the perceptions and attitude of Egyptian physicians towards ethical issues in biobanking.<br><br>Methods : A cross-sectional online survey was designed and communicated with the target group between November 2019 and January 2020.<br><br>Results : The questionnaire was completed by 223 physicians. While 65.5% reported hearing the term "Biobanking" before, 45.7% knew that there are biobanks in Egypt. Participants had a general positive attitude towards the value of biobanks in research. About 73% agreed that biobanks can share samples with international research organizations, but only 42.6% supported collaboration with pharmaceutical companies, and 44% agreed to the use of user fees by biobanks. About 48% supported the use of broad consent in biobanks, and 73.1% believed that sample donors should be informed about results of research performed on their samples.<br><br>Conclusion : Although many Egyptian physicians heard about biobanking, they had limited knowledge about the existence of biobanks in Egypt. They had concerns about commercialization, use of broad consent and user fees. A knowledge gap exists among these stakeholders, which should be covered by different educational activities. Community discussions should start to reach consensus about the issues of commercialization and return of research results
**Order of Authors:**	Ahmed Samir Abdelhafiz, PhD
	Eman A. Sultan
	Hany H. Ziady
	Douaa M. Sayed
	Walaa A. Khairy
**Additional Information:**	
**Question**	**Response**
**Financial Disclosure**<br><br>Enter a financial disclosure statement that describes the sources of funding for the work included in this submission. Review	The author(s) received no specific funding for this work.

the submission guidelines for detailed requirements. View published research articles from *PLOS ONE* for specific examples.<br><br>This statement is required for submission and **will appear in the published article** if the submission is accepted. Please make sure it is accurate.<br><br>**Unfunded studies**<br>Enter: *The author(s) received no specific funding for this work.*<br><br>**Funded studies**<br>Enter a statement with the following details:<br>• Initials of the authors who received each award<br>• Grant numbers awarded to each author<br>• The full name of each funder<br>• URL of each funder website<br>• Did the sponsors or funders play any role in the study design, data collection and analysis, decision to publish, or preparation of the manuscript?<br>• **NO** - Include this sentence at the end of your statement: *The funders had no role in study design, data collection and analysis, decision to publish, or preparation of the manuscript.*<br>• **YES** - Specify the role(s) played.<br><br>* typeset	
**Competing Interests**<br><br>Use the instructions below to enter a competing interest statement for this submission. On behalf of all authors, disclose any competing interests that could be perceived to bias this work—acknowledging all financial support and any other relevant financial or non-financial competing interests.<br><br>This statement **will appear in the published article** if the submission is accepted. Please make sure it is accurate. View published research articles from *PLOS ONE* for specific examples.	The authors have declared that no competing interests exist.

**NO authors have competing interests**<br><br>Enter: *The authors have declared that no competing interests exist.*<br><br>**Authors with competing interests**<br><br>Enter competing interest details beginning with this statement:<br><br>*I have read the journal's policy and the authors of this manuscript have the following competing interests: [insert competing interests here]*<br><br>* typeset	
**Ethics Statement**<br><br>Enter an ethics statement for this submission. This statement is required if the study involved:<br><br>• Human participants<br>• Human specimens or tissue<br>• Vertebrate animals or cephalopods<br>• Vertebrate embryos or tissues<br>• Field research<br><br>Write "N/A" if the submission does not require an ethics statement.<br><br>General guidance is provided below. Consult the submission guidelines for detailed instructions. **Make sure that all information entered here is included in the Methods section of the manuscript.**	Ethical Committee of Alexandria Faculty of Medicine. Approval number 0304618

**Format for specific study types**<br><br>**Human Subject Research (involving human participants and/or tissue)**<br>• Give the name of the institutional review board or ethics committee that approved the study<br>• Include the approval number and/or a statement indicating approval of this research<br>• Indicate the form of consent obtained (written/oral) or the reason that consent was not obtained (e.g. the data were analyzed anonymously)<br><br>**Animal Research (involving vertebrate animals, embryos or tissues)**<br>• Provide the name of the Institutional Animal Care and Use Committee (IACUC) or other relevant ethics board that reviewed the study protocol, and indicate whether they approved this research or granted a formal waiver of ethical approval<br>• Include an approval number if one was obtained<br>• If the study involved *non-human primates*, add *additional details* about animal welfare and steps taken to ameliorate suffering<br>• If anesthesia, euthanasia, or any kind of animal sacrifice is part of the study, include briefly which substances and/or methods were applied<br><br>**Field Research**<br><br>Include the following details if this study involves the collection of plant, animal, or other materials from a natural setting:<br>• Field permit number<br>• Name of the institution or relevant body that granted permission	
**Data Availability**<br><br>Authors are required to make all data underlying the findings described fully available, without restriction, and from the time of publication. PLOS allows rare exceptions to address legal and ethical concerns. See the PLOS Data Policy and FAQ for detailed information.	Yes - all data are fully available without restriction

A Data Availability Statement describing where the data can be found is required at submission. Your answers to this question constitute the Data Availability Statement and **will be published in the article**, if accepted.<br><br>**Important:** Stating 'data available on request from the author' is not sufficient. If your data are only available upon request, select 'No' for the first question and explain your exceptional situation in the text box.<br><br>Do the authors confirm that all data underlying the findings described in their manuscript are fully available without restriction?	
**Describe where the data may be found in full sentences. If you are copying our sample text, replace any instances of XXX with the appropriate details.**<br><br>• If the data are **held or will be held in a public repository**, include URLs, accession numbers or DOIs. If this information will only be available after acceptance, indicate this by ticking the box below. For example: *All XXX files are available from the XXX database (accession number(s) XXX, XXX.)*.<br>• If the data are all contained **within the manuscript and/or Supporting Information files**, enter the following: *All relevant data are within the manuscript and its Supporting Information files.*<br>• If neither of these applies but you are able to provide **details of access elsewhere**, with or without limitations, please do so. For example:<br><br>*Data cannot be shared publicly because of [XXX]. Data are available from the XXX Institutional Data Access / Ethics Committee (contact via XXX) for researchers who meet the criteria for access to confidential data.*<br><br>*The data underlying the results presented in the study are available from (include the name of the third party*	All relevant data are within the manuscript and its Supporting Information files.

and contact information or URL).<br>• This text is appropriate if the data are owned by a third party and authors do not have permission to share the data.<br><br>* typeset	
Additional data availability information:	

Manuscript

Click here to access/download;Manuscript;What Egyptians think doctors with tables 30-10.docx

Knowledge, Perceptions and Attitude of Egyptian Physicians towards Biobanking Issues

Ahmed S. Abdelhafiz[1], Eman A. Sultan[2], Hany H. Ziady[2], Douaa M. Sayed[3], Walaa A. Khairy[4]

1. Department of Clinical pathology, National Cancer Institute, Cairo University, Cairo, Egypt.
2. Department of Community Medicine, Faculty of Medicine, Alexandria University, Alexandria, Egypt.
3. Department of Clinical pathology, South Egypt Cancer Institute, Assiut University, Assiut, Egypt.
4. Department of Community Medicine, Faculty of Medicine, Cairo University, Cairo, Egypt.

Corresponding author: Ahmed S. Abdelhafiz. Email: ahmed.samir@nci.cu.edu.eg

Address: National Cancer Institute, Cairo University, Kasr Al-Aini Street, Fom Elkhalig square, Cairo, Egypt.

Postal Code: 11796

Mobile no: +201097781290

Abstract

Objectives: Collection and storage of biospecimens and data for biobanking raise many ethical concerns. Stakeholders' opinions about these ethical issues are important since they can help in the development of ethical guidelines to govern biobanking activities. Physicians are among the important stakeholders since they contact potential participants and could be biobank users. The goal of this study is to evaluate the perceptions and attitude of Egyptian physicians towards ethical issues in biobanking.

Methods: A cross-sectional online survey was designed and communicated with the target group between November 2019 and January 2020.

Results: The questionnaire was completed by 223 physicians. While 65.5% reported hearing the term "Biobanking" before, 45.7% knew that there are biobanks in Egypt. Participants had a general positive attitude towards the value of biobanks in research. About 73% agreed that biobanks can share samples with international research organizations, but only 42.6% supported collaboration with pharmaceutical companies, and 44% agreed to the use of user fees by biobanks. About 48% supported the use of broad consent in biobanks, and 73.1% believed that sample donors should be informed about results of research performed on their samples.

Conclusion: Although many Egyptian physicians heard about biobanking, they had limited knowledge about the existence of biobanks in Egypt. They had concerns about commercialization, use of broad consent and user fees. A knowledge gap exists among these stakeholders, which should be covered by different educational

activities. Community discussions should start to reach consensus about the issues of commercialization and return of research results.

Keywords: Biobanking; Egypt; Physicians; Perception; Attitude

Introduction

The complete mapping of the human genome brought many possibilities to understand human diseases and to improve their management, announcing a new era for personalized medicine (1,2). This development was associated with the need for the development of biobanks, which can supply large amounts of samples and data required to conduct large genomic studies (3).

Both genomic research and population biobanks are growing worldwide, guided by the hope that this research will lead to better disease prevention and treatment; through novel drug targeting, personalized treatment, as well as prediction of disease risk (1,2,4,5). In addition to population based biobanks, human biobanks can be disease based (collect samples from patients with a specific disease or a group of related diseases). They can be non profit, commercial, or even virtual biobanks (An electronic database of biospecimens and data that exists independent of the location of the stored samples) (5-8).

One of the biggest challenges for biobanks is to achieve long-term sustainability (9). Social, operational, as well as financial aspects should be taken into consideration when thinking about sustainability of biobanks (10,11). While operational sustainability focuses on the efficiency of the operations of the biobank, social sustainability focuses on the acceptability of the biobank and its activities by stakeholders through proper engagement of these stakeholders (10).

Stakeholders can have a strong influence on biobanks, and their satisfaction is one of the keys to biobanks success. Biobank stakeholders include participants, physicians, researchers, research institutions, funders, as well as ethics committees

(2,9). It is important to have a strategy for identifying, communication and engagement with stakeholders to achieve mutual benefits (10).

Major changes in the research environment, including the growth of multi-institutional projects, together with sharing of large amounts of data, and advances in genomic technologies, have raised new ethical issues (12). These include, among others, issues about privacy and confidentiality, protection of human subjects, benefit sharing, commercialization and genomic data sharing (12).

In Egypt, eight disease based biobanks have been established in the past few years to support clinical and genomic research (13). These biobanks are distributed in six governorates form the north (Alexandria), to the south (Luxor and Aswan) of Egypt. All of them are nonprofit and affiliated to universities or nongovernmental organizations. Although their number and activities is growing, there are no specific guidelines or regulations for biobanks; only few medical documents containing chapters about clinical research exist in Egypt (14). The development of local ethical guidelines to govern biobanking should take the opinions of different stakeholders into consideration, and the first step of building a good relationship with different stakeholders is to understand their knowledge and attitude towards the biobanking concept and related issues.

In our previous work, we studied these issues among Egyptian patients (7). In the current work, we present the results of a survey conducted with physicians to assess their knowledge, perceptions and attitudes towards biobanking issues.

Subjects and methods

Study design, Participants, Sampling and Setting

A cross-sectional online survey was designed to implement the study. The target population was Egyptian physicians currently working in Egypt at different

public and private health centers representing the three main geographical regions of Egypt; the north (Alexandria), the capital or the center (Cairo), and the south (Assiut). The physicians were recruited through an online Google form. The sample size was determined using the Epi Info 7 software based on the expected probability of positive attitude of health professionals towards biobanking (82.9%) (15) at 95% confidence interval, limit of precision of 5%, with a design effect of 1.0. The calculated sample size was 218 participants.

Study tool and data collection

An online questionnaire was developed in English. The survey started with an introduction explaining what are biobanks and their role in research, followed by a few links to watch and read more about biobanks (Supplementary files). Based on this information, and after a section on sociodemographic data, the questionnaire included questions about basic knowledge about biobanking before the time of the survey, followed by questions about perceptions and attitudes towards different issues related to biobanking.

A preliminary phase was conducted to assess validity and reliability of the questionnaire before its use. For assessment of validity, three Egyptian experts in the field of biomedical research were asked to assess the degree to which items in the questionnaires were relevant and could correctly measure the knowledge and attitudes of researchers about biobanking. After that, corrections were done as per the experts' remarks. For assessment of reliability, 15 physicians were asked to respond to the questionnaire twice three weeks apart. The results showed adequacy of both internal consistency reliability (Cronbach's alpha =0.78), and test-retest reliability (intra-class correlation coefficient = 0.97) (16).

During the data collection phase, the investigators sent invitation links by email to physicians using the convenient sample technique. Facebook and other social media platforms were also used to recruit participants. A four-month period was required to achieve the required sample size. The data were collected between November 2019 and January 2020.

Statistical analysis

All data analyses were performed using Statistical Package for the Social Sciences (SPSS) software, version 22. Data were presented as numbers and percentages for categorical variables, and as means and standard deviations (SD) for quantitative continuous variables. For the purpose of testing associations between qualitative variables, the chi-squared test was used. All results were interpreted at the 5% level of significance.

Ethical considerations

The study was performed in accordance with relevant regulations (17). A statement saying "By completing this questionnaire, you agree to participate with us in this study" was considered as an informed consent to participate in the study. Official approvals for implementation of the study were obtained from the Ethical Committee of Alexandria Faculty of Medicine. Confidentiality of data was ensured all through the phases of the study and continued till the full manuscript was finalized.

Results

The questionnaire was completed and submitted by 223 out of 291 physicians, yielding a response rate of 76.6%. About half of participants (47.1%) were from Cairo, followed by Alexandria (37.7%), and Assiut (15.2%). The demographic and biobanking related characteristics of the respondents are summarized in supplementary table 1. Nearly half the respondents (43%) reported that they were

working/ planning to work on blood samples for their current/future research, while only 38.1% and 25.1% of the respondents were working/ planning to work on tissue and saliva/urine samples, respectively. Most of the physicians (79.4%) didn't attend any type of scientific activity about biobanking.

The physicians' responses to the basic knowledge items about biobanking are displayed in table 1. About two thirds of the physicians reported hearing the term "biobanking" before. However, only 45.7% thought that there are several biobanks in Egypt.

Table 1: Basic knowledge about Biobanking among the respondents (n = 223)

Knowledge item	Number	Percent
**I have heard the term "Biobanking" before**	146	65.5
**There is a biobank in my institute**	69	30.9
**There are several biobanks in Egypt**	102	45.7
**There is a law that governs biobank work in Egypt**	114	51.1

Table 2 shows the physicians' general attitudes towards biobanking. The majority of the participants' responses towards the survey items expressed positive attitudes towards biobanking (strongly agree – agree). Noteworthy, physicians recorded the highest level of agreement (strongly agree – agree) regarding the following statements "*I would like more information about biobanks to be more readily available*" (97.3%); "*I think biobanks can make a difference in biomedical research in general*" (95.9%); "*The presence of biobanks is important for the development of new treatments*" (95.5%); and "*The presence of biobanks is important for the development of new methods of diagnosis*" (94.1%). Moreover, the following statements showed the highest level of uncertainty (unsure) among the researchers *"I*

will donate samples myself and will ask my relatives to donate samples to the biobank" (39%); "*Donating samples for research is in line with religious beliefs*" (30%); "*I would be willing to help create a biobank in my institute*" (21.1%); and "*In the future, I will be interested in applying to get samples for my research from the biobank*" (20.6%).

Table 2: Level of agreement of the respondents to the survey items regarding general attitudes towards biobanking (n = 223)

Survey Items	Level of Agreement									
	Strongly Agree		Agree		Unsure		Disagree		Strongly Disagree	
	N	%	N	%	N	%	N	%	N	%
1. I think that the presence of biobanks is important for the development of new treatments	115	51.6	98	43.9	9	4.0	0	0.0	1	0.4
2. I think that the presence of biobanks is important for the development of new methods of diagnosis	114	51.1	96	43.0	12	5.4	0	0.0	1	0.4
3. I think biobanks can make a difference in biomedical research in general	116	52.0	98	43.9	8	3.6	0	0.0	1	0.4
4. I think biobanks can make a difference in aspects related to quality of samples and data provided for research	104	46.6	102	45.7	16	7.2	0	0.0	1	0.4
5. In the future, I will be interested in applying to get samples for my research from the biobank	78	35.0	96	43.0	46	20.6	2	0.9	1	0.4
6. I would be willing to help create a biobank in my institute	81	36.3	91	40.8	47	21.1	1	0.4	3	1.3
7. I will donate samples myself and will ask my relatives to donate samples to the biobank	45	20.2	79	35.4	87	39.0	8	3.6	4	1.8
8. If there is a biobank in my institution, I will inform my patients about it	80	35.9	108	48.4	31	13.9	2	0.9	2	0.9
9. I would like more information about biobanks to be more readily available	141	63.2	76	34.1	5	2.2	0	0.0	1	0.4
10. If there is a course/ lecture/conference about Biobanking, I will be interested in attending	77	34.5	113	50.7	31	13.9	0	0.0	2	0.9
11. Donating samples for research is in line with religious beliefs	61	27.4	88	39.5	67	30.0	4	1.8	3	1.3

Table 3 reveals the participants' responses to privacy, sharing and access issues. Most of the responses revealed concerns about these survey items. The highest level of disagreement (strongly disagree – disagree) and uncertainty (unsure) were attained by the following statements "*I will not donate my samples to a biobank because my identity could be known through my DNA*", where 41.3% didn't agree to this statement, and 38.6% were not sure about it. The same applied for "*The biobank may provide any medical information to insurance companies*" where 30.0 % were unsure and 38.1% didn't agree with statement; and for "*Biobanks can share samples and data with commercial and pharmaceutical companies*" (26.9% unsure, and 30.5% disagree); and "*The biobank may provide any medical information to the government*" (27.8% unsure, and 27.3% disagree). On the other hand, the following statements revealed the highest level of agreement (strongly agree – agree) "*The biobank may provide any medical information to treating physician*" (82.1%); and "*Biobanks may share samples and data with international research organizations*" (73.1%).

Table 3: Level of agreement of the respondents to the survey items regarding privacy, sharing and access issues (n = 223)

Survey Items	Level of Agreement									
	Strongly Agree		Agree		Unsure		Disagree		Strongly Disagree	
	N	%	N	%	N	%	N	%	N	%
1. Biobanks can share samples and data with international research organizations	62	27.8	101	45.3	39	17.5	13	5.8	8	3.6
2. Biobanks can share samples and data with commercial and pharmaceutical companies	24	10.8	71	31.8	60	26.9	38	17.0	30	13.5
3. The biobank may provide any medical information to insurance companies	16	7.2	55	24.7	67	30.0	50	22.4	35	15.7
4. The biobank may provide any medical information to treating physician	56	25.1	127	57.0	29	13.0	6	2.7	5	2.2
5. The biobank can provide any medical information to government	23	10.3	77	34.5	62	27.8	34	15.2	27	12.1
6. The biobank can provide confidential medical information to legal authorities if asked	28	12.6	97	43.5	48	21.5	28	12.6	22	9.9

7. **I will not donate my samples to a biobank because my identity could be known through my DNA**	10	4.5	35	15.7	86	38.6	74	33.2	18	8.1

The level of agreement of respondents to governance issues is presented in table 4. Out of five survey items, the majority of the physicians (94.2%) supported (strongly agree – agree) this item "*A transparent policy for distribution of samples to researchers should exist*".

Table 4: Level of agreement of the respondents to the survey items regarding governance (n = 223)

**Survey Items**	**Level of Agreement**									
	**Strongly Agree**		**Agree**		**Unsure**		**Disagree**		**Strongly Disagree**	
	**N**	**%**	**N**	**%**	**N**	**%**	**N**	**%**	**N**	**%**
1. **A participant who donates blood for scientific research on genes and environment remains in control over his/her blood**	22	9.9	81	36.3	72	32.3	42	18.8	6	2.7
2. **Biobanks owns the stored samples**	14	6.3	65	29.1	71	31.8	59	26.5	14	6.3
3. **Biobanks are just in custody of the samples, but don't own them.**	34	15.2	108	48.4	50	22.4	28	12.6	3	1.3
4. **Biobanks may charge user fees for samples that are distributed to researchers.**	12	5.4	86	38.6	63	28.3	45	20.2	17	7.6
5. **A transparent policy for distribution of samples to researchers should exist.**	124	55.6	86	38.6	10	4.5	1	0.4	2	0.9

Regarding the consent and participant's rights, most of the responses related to these issues revealed agreement among the physicians as displayed in table 5. The following survey items yielded the highest level of agreement (strongly agree – agree) "*Participants' information and samples stored in the biobank should be protected and securely stored*" (96%); "*Sample donors should be informed in detail how their samples will be used*" (84.8%); and "*Sample donors should be informed if their samples will be transferred abroad*" (78.1%).

Table 5: Level of agreement of the respondents to the survey items regarding consent and participant's rights (n = 223)

Survey Items	Level of Agreement									
	Strongly Agree		Agree		Unsure		Disagree		Strongly Disagree	
	N	%	N	%	N	%	N	%	N	%
**1. A broad consent that doesn't include every future research is more suitable for biobank work.**	34	15.2	73	32.7	82	36.8	24	10.8	10	4.5
**2. Participant information and samples stored in the biobank should be protected and securely stored.**	159	71.3	55	24.7	6	2.7	1	0.4	2	0.9
**3. Sample donors should be compensated by some means for their samples**	28	12.6	80	35.9	68	30.5	40	17.9	7	3.1
**4. Sample donors should be informed in detail how their samples will be used**	105	47.1	84	37.7	19	8.5	11	4.9	4	1.8
**5. Sample donors should be informed if their samples will be transferred abroad**	111	49.8	63	28.3	32	14.3	14	6.3	3	1.3
**6. Sample donors should be informed about results resulting from research on their samples**	82	36.8	81	36.3	44	19.7	16	7.2	0	0.0

The associations between demographic data and biobanking related variables and the knowledge about biobanking are summarized in supplementary tables 2 and 3. Hearing the term "Biobank" before was significantly associated with two variables namely; gender (P=0.019) and attending any scientific activity about biobanking before (P<0.001). No significant association was found between knowledge about the term before and age, residence, years of experience, affiliation, or specialty. There was no significant association between knowledge about biobanking and the type of research of current or future research of the participant.

Discussion

Human biobanks have shown rapid expansion in the past 20 years. Current genomic research is based on using large numbers of samples from many participants

to reach statistically significant conclusions for healthcare (6). In order to play their role in research, biobanks should have an effective governance framework. This framework includes the rules and practices by which the biobank ensures accountability, fairness, and transparency to funders, patients, researchers, as well as the society. Development of stakeholder engagement strategies is an important component of biobank governance (18). Physicians are among the important biobank stakeholders due to several reasons. First, they communicate with the patients, get their trust, can give them information about research and ask for their permission to be contacted by biobank personnel (19). Second, most physicians in Egypt must engage in research activity in the form of a master and/or PhD to get their specializations. Therefore, they can be users of biobank services. Finally, currently all biobanks in Egypt are disease-based. Thus, it is important to engage physicians, and study their perceptions and attitude towards biobanking issues, including ethical issues, which is the goal of this study.

1. **Knowledge about biobanking**

In general, our participants had an acceptable level of knowledge about the existence of biobanks (Table 1). In our previous study, more than eighty percent of patients participating in a similar survey reported that they never heard about the "Biobanking" term before (7). This makes sense, since physicians are more involved in research and are familiar with its terms. A previous study showed that there was a lower level of knowledge (36%) about biobanking among health professionals in Australia (20). This difference may be attributed to the difference in the study population, since most health professionals in the aforementioned study were nurses. However, our participants had limited knowledge about the existence of biobanks in

Egypt, which reflects the limited marketing activities used by these biobanks to communicate with these stakeholders. This was also evident from the limited number of participates who attended any educational activities about biobanking (Supplementary table 1). Knowledge about the term "biobanking" was significantly higher among those who attended these scientific events before (Supplementary table 3). In our previous work, we used Facebook, the most popular social media platform in Egypt, successfully to communicate the biobanking concept with undergraduate students of life sciences (13). That's why we recommend using social media platforms for marketing the biobanking concept with physicians as well, since they have proven effective in communicating with different stakeholders about biobanking and health issues in Egypt (13,21).

2. General attitude towards biobanking

In general, our participants had a positive attitude towards biobanking and recognized their value for biomedical research (Table 2). This positive attitude is similar to that reported among health professionals in the study conducted with health professionals in Australia (20). However, only 55.6% of participants were willing to donate their own samples or ask their family members to donate samples to the biobanks. Interestingly, a similar proportion (57.7%) of Egyptian medical students participating in similar survey had the same response (data not published). We think that as the educational level increases, willingness to donate samples decreases. This is similar to the study conducted by the biobank team in Children’s Cancer Hospital Egypt, who reported a negative correlation between willingness to donate samples to the biobank and higher levels of education (22). Some physicians, as well as medical students, may think that they should lead the process of patient care and research, but

do not prefer to be a part of it. They may also have concerns or fears regarding participation in genetic research. These concerns should be explored in future studies, and respected while approaching them.

Islam is the first religion in Egypt, and the second article of the Egyptian constitution states that "“Islam is the state religion, and that principles of Islamic Jurisprudence are the main source of legislation" (23). In our study, about 30% of participants were not sure about the opinion of their religion regarding sample donation (Table 2). This in contrast to patients in our previous survey, where 86.5% believed that sample donation did not contradict their religious beliefs. This might reflect the nature of physicians who look for evidence based opinions in their practice as well as their lives. Muslim religious scholars have indicated that Islam permits the establishment of research biobanks if autonomy is respected (24,25). In general, confidentiality has received much attention in Islamic resources including the Qur'an, Sunnah (Habits, practices, words, and decisions of the Prophet Muhammad), and juristic writings (24). The website of Qatar biobank, which is another Islamic country, states that "Islam is permissive when it comes to the use of biological information for research purposes if it is regulated within the context of culture and religion"(26). Collectively, we do not think that religion will represent a barrier for the establishment and work of biobanks in Egypt if the general values of research ethics are respected.

3. **Perception and attitude regarding privacy, access, and sharing of samples**

Biobanks collect and store samples and data to provide them for researchers for use in different research projects. Strong governance and transparent policies of

access to these collections should be present (27). In general, access to samples and data should be governed according to scientific quality, value and ethical soundness (27). The governance of access is challenged by the different and sometimes conflicting interests of stakeholders, including biobank managers (who may seek academic or financial recognition of their efforts when material or data are used in research), researchers who pursue their own research interests when they ask for access for samples and data, and funders (who may oblige biobanks to permit the uses of samples and data in research with high scientific and social value only) (27).

Sharing of samples across borders and with commercial entities raises even more concerns. Although sharing of different types of samples and data is now a routine in international collaborative research, it is associated with many challenges, including benefit sharing, intellectual property rights, as well as authorship over publications (28,29). Trassport of samples across borders is even more complicated in Egypt, where a law about clinical research has put restrcitions over sample transport across borders (30). The approved law by the parliament, was later on rejected by the prisedent, who objected to some articles (31).

Most participants in our survey, and in contrast to patients in our previous survey (7), supported sample and data sharing with international research organizations (Table 3). However, collaboration with commercial and pharmaceutical companies represented a concern for them, where 42.6% only agreed to samples and data sharing with commercial and pharmaceutical companies. Although commercialization represents one of the means to achieve financial sustainability (11), it raises ethical question about fair sharing of benefits, and represents a concern for biobank sample donors (32). In our previous study, less than 28% of our survey participants supported sharing their potential samples with pharmaceutical companies

(7). Taken together, we think that several stakeholders in Egypt are not ready to deal with the issue of commercialization due to fears about misuse of their samples and data. That's why we recommend that of commercialization of biobanks in Egypt should be delayed until these concerns are discussed and dealt with. We also think that sample and data sharing across borders in Egypt should be regulated by the law, which should balance the benefits with the risks of sharing.

A balance should be also be maintained between protecting privacy and confidentiality from one side, and the benefits for the individual and/or the society on the other side. Our participants provided a framework for this point, where most of them agreed that the biobank can give access of medical information to treating physician when needed, but not to the government or to insurance companies. On the other hand, a bit more than 50% of them thought that the biobank can provide confidential medical information to legal authorities if asked (Table 3). In our study with patients, 71.8% of participants agreed on a similar question (7). The majority of participants in a survey conducted with different stakeholders in Saudi Arabia, which included researchers, physicians, medical students, donors and laypersons, agreed that confidentiality might be breached in cases with specific justified reasons. These conditions included the presence of infectious and genetic disease, court order, as well as other reasons (33). In a study that aimed at exploring stakeholder perspectives on the use of biological samples for future unspecified research in Malawi, participants highlighted the importance of privacy and confidentiality, and believed that it should be a top priority. They also reported that there is need for guidelines for sharing and access to samples (34). Recently, the Egyptian president endorsed a law on personal data protection, which meant to promote the security of personal data, which is being processed and stored online. It also sets a legal framework to regulate data

transmission with other countries (35). We think that there should be clear justifications for any breach of privacy if samples were to be used for purposes other than research, and this should be regulated by law.

4. Perceptions and attitude issues related to governance of biobanks

Millions of human samples are collected and stored in biobanks. The long term storage of these samples and associated data raises questions about control and ownership of the samples (36). Most participants in our survey thought that biobanks are just in custody of the samples, but do not own them (Table 4). A study conducted to explore the views of university researchers' in the United States on ownership of human genetic specimens, showed that they had divergent views on ownership of retained specimens, and many of them identified more than one owner (36)

A previous study proposed that "custodianship" best describes the role of researchers and biobanks with regard to samples (37). Custodianship is the responsibility for caretaking of biospecimens from initial collection till distribution of research findings, where practices and operating principles for responsible oversight of samples collected for research are endorsed (37). Custodial model ensures transparency in research, justice for research participants, and accountability among all stakeholders involved in biospecimen research (37). Another study proposed "stewardship" model, which implies that all members of the research team are responsible for protection of participants' interests and well-being to the best of their abilities (38). We agree that the concepts; custody and stewardships, are better than ownership to describe the role of the biobanks with regards to samples.

To support the growing need for high quality biospecimens and data, biobanks should have a business plan to achieve operational, social and financial sustainability

over the intended duration of the bioabnk. Financial sustainability may be achieved through institutional support, together with short and long term grants (11). However, these methods are not always available, and biobanks should consider cost recovery models and strategies, including biospecimen user fees for access to human specimens, data, and sales of services provided by the biobanks (39). The concept of user fees is different from commercialization, which raises a lot of debate (40). Less than 50% of medical doctors participating in our survey agreed to the concept of user fees. We think that the majority of them confused user fees with selling/commercialization of samples, which reflects the limited knowledge about this point.

5. **Perceptions regarding consent and participant's rights**

Biobanks store biospecimens and data for future unspecified research projects. Appropriate consent for sample donors must explain the scope and breadth of consent together with different elements of the biobank governance framework (41). Several types of consent can be used by the biobank. These include, among others, specific consent (specific for a single research project), dynamic consent (using technology to allow participants to choose between broad consent or to approve a single study at a time), and tiered consent (where the participant allows some uses of the samples only and renewal of consent is needed for other studies (12,40,42). A broad consent that contains adequate details is a suitable model of consent for use in biobanks (43). It should be noted that broad consent is not equivalent to blanket consent, in which there are no details about the use of samples. Broad consent should contain details about the nature of the biobank, type of samples and data that will be collected, privacy, access, possibility of re-contact, sharing conditions and benefit sharing, as wells as commercialization of the samples (28,44,45,46).

In our study, we chose to ask participants about broad consent only for two reasons. First, we think that it is the best consent model for adoption in Egypt, being flexible and can be used with minimal costs. Second, it is the consent model adopted already by different biobanks in Egypt. In our survey, less than fifty percent of our participants agreed to the use of broad consent in biobanks (Table 5). We think that the majority of our participants did not know about the existence of this type of consent, and assumed that this contradicts the right of sample donors to know how their samples will be used. Again this reflects the knowledge gap, which should be filled by biobankers in Egypt.

Another potential right is the right of sample donors to know results from research performed on their samples (47). The majority of participants in this survey, like patients in our previous study (7), believed in this right. A survey conducted to assess the opinions of stakeholders regarding the return of individual research results among researchers, physicians, and laypeople in Saudi Arabia showed that there was a strong agreement among participants that sample donors have the right to get results that may have direct or indirect benefits if they want (48).

Return of research results and incidental findings is complicated and raises a lot of ethical questions. Examples of these questions are what should be returned and considered as ‘actionable’’, and what should be considered as nonbeneficial results (48). Another debate is related to how these results, which may include complex genetic results, will be explained to participants and how it will affect their insurance, employment or health care (49). The majority of Saudi health care professionals participating in a study about this issue agreed on the importance of returning research results, and highlighted a number of challenges that may be experienced during returning results generated during the process of research. These challenges included

challenges related to the donor (Such as the psychological state and difficult communication), and challenges related to information (such as certainty about the results and cost of returning results (47). We recommend the establishment of a dialogue about these issues among concerned parties in Egypt, together with proper training of genetic councilors on how to communicate these results with participants/patients.

Conclusions and recommendations

Although Egyptian physicians had an acceptable level of knowledge about biobanking, they had limited knowledge about the existence of biobanks in Egypt. They had concerns about commercialization of biobanks, the use of broad consent and user fees. A knowledge gap exists among these important biobanks stakeholders, which should be covered by active marketing and engagement activities by biobankers in Egypt. Broad community discussions should start to reach consensus about the issues of commercialization and return of research results.

Limitations of the study

The collection of data from physicians from three governorates only, and the lack of knowledge about participants' education about bioethics before the survey represent limitations to this study.

Funding

No source of external funding was received for this research

Competing Interests

The authors have no conflicts of interest to disclose.

References

1. Yakubu, A., Tindana, P., Matimba, A., et al. (2018). Model framework for governance of genomic research and biobanking in Africa - a content description. *AAS open research*, *1*, 13. https://doi.org/10.12688/aasopenres.12844.1
2. Swede, H., Stone, C. & Norwood, A. National population-based biobanks for genetic research. *Genet Med* **9,** 141–149 (2007). https://doi.org/10.1097/GIM.0b013e3180330039
3. Amin, L., Hashim, H., Mahadi, Z., et al. (2018). Determinants of the willingness to participate in biobanking among Malaysian stakeholders in the Klang Valley. *BMC medical research methodology*, *18*(1), 163. https://doi.org/10.1186/s12874-018-0619-2
4. Cambon-Thomsen, A., Ducournau, P., Gourraud, P. A., et al. (2003). Biobanks for genomics and genomics for biobanks. *Comparative and functional genomics*, *4*(6), 628–634. https://doi.org/10.1002/cfg.333
5. Thompson, R., & McNamee, M. J. (2017). Consent, ethics and genetic biobanks: the case of the Athlome project. *BMC genomics*, *18*(Suppl 8), 830. https://doi.org/10.1186/s12864-017-4189-1
6. Husedzinovic, A., Ose, D., Schickhardt, C., Fröhling, S., et al. (2015). Stakeholders' perspectives on biobank-based genomic research: Systematic review of the literature. *European Journal of Human Genetics*, *23*(12), 1607–1614. https://doi.org/10.1038/ejhg.2015.27
7. Abdelhafiz, A.S., Sultan, E.A., Ziady, H.H. *et al.* What Egyptians think. Knowledge, attitude, and opinions of Egyptian patients towards biobanking

issues. *BMC Med Ethics* **20,** 57 (2019). https://doi.org/10.1186/s12910-019-0394-6

8. van Draanen, J., Davidson, P., Bour-Jordan, H., et al. (2017). Assessing Researcher Needs for a Virtual Biobank. Biopreservation and biobanking, 15(3), 203–210. https://doi.org/10.1089/bio.2016.0009

9. Bjugn, R., & Casati, B. (2012). Stakeholder analysis: A useful tool for biobank planning. Biopreservation and Biobanking, 10(3), 239–244. https://doi.org/10.1089/bio.2011.0047
10. Simeon-Dubach, D., & Henderson, M. K. (2014). Sustainability in Biobanking. Biopreservation and Biobanking, 12(5), 287–291. https://doi.org/10.1089/bio.2014.1251
11. Campos AH, Schreeder M, Parry-Jones A, et al. Addressing the Challenge of Financial Sustainability in Biobanking. Biopreserv Biobank. 2015;13(6):387-395. doi:10.1089/bio.2015.29042.ahc.
12. Bledsoe MJ. Ethical Legal and Social Issues of Biobanking: Past, Present, and Future. Biopreserv Biobank. 2017;15(2):142-147. doi:10.1089/bio.2017.0030
13. Abdelhafiz AS, Fouda MA, El-Jaafary SI, et al. Targeting future customers: an introductory biobanking course for undergraduate students of life sciences. Biopreserv Biobank. 2017;15(4):350–9. https://doi.org/10.1089/bio.2016.0111.
14. Alahmad G, Al-Jumah M, Dierickx K. Review of national research ethics regulations and guidelines in Middle Eastern Arab countries. BMC Med Ethics. 2012 Dec 12;13:34. doi: 10.1186/1472-6939-13-34. PMID: 23234422; PMCID: PMC3576357

15. Lhousni S, Boulouiz S, Abda N, et al. (2019). Assessment of Knowledge, Attitudes and Support of Health Professionals towards Biobanks in Eastern Morocco. Open Journal of Epidemiology 2019; 9: 191-201
16. Wells KJ, Arevalo M, Meade CD, et al. Development and validation of the biobanking attitudes and knowledge survey (BANKS). Cancer Epidemiol Biomarkers Prev. 2014;23(3):374-382. doi:10.1158/1055-9965.EPI-13-0746
17. World Medical Association. World Medical Association Declaration of Helsinki: ethical principles for medical research involving human subjects. JAMA. 2013;310(20):2191-2194. doi:10.1001/jama.2013.281053.
18. Murphy, M., Garrett, S. B., Boyd, E., et al.. (2017). Engaging Diverse Stakeholders to Inform Biobank Governance. Biopreservation and biobanking, 15(4), 393–395.https://doi.org/10.1089/bio.2016.0061
19. Cheah S, O'Donoghue S, Daudt H, et al. Permission to contact (PTC) a strategy to enhance patient engagement in translational research. Biopreserv Biobank. 2013;11(4):245–252. doi:10.1089/bio.2013.0023
20. Caixeiro, N. J., Byun, H. L., Descallar, J., et al.. (2016). Health professionals' opinions on supporting a cancer biobank: identification of barriers to combat biobanking pitfalls. European journal of human genetics : EJHG, 24(5), 626–632. https://doi.org/10.1038/ejhg.2015.191
21. Abdelhafiz, A.S., Mohammed, Z., Ibrahim, M.E. et al. Knowledge, Perceptions, and Attitude of Egyptians Towards the Novel Coronavirus Disease (COVID-19). J Community Health (2020). https://doi.org/10.1007/s10900-020-00827-7
22. Labib RM, Hassanain O, Alaa M, et al. Planning today for Tomorrow's research: analysis of factors influencing participation in a pediatric cancer research

biorepository. Front Oncol. 2018;7 (January):1–6. https://doi.org/10.3389/fonc.2017.00324.

23. The Egyptian Constitution 2014 Available online at https://bit.ly/2T3RP8C (Accessed 7 August, 2020)
24. Alahmad G, Dierickx K. What do Islamic institutional fatwas say about medical and research confidentiality and breach of confidentiality? Dev World Bioeth. 2012;12(2):104–12. https://doi.org/10.1111/j.1471- 8847.2012.00329.
25. Alahmad G, Dierickx K. Ethics of research biobanks: Islamic perspectives. Biopreserv Biobank. 2018. https://doi.org/10.1089/bio.2017.0067
26. Qatar biobank, Frequently Asked Questions, Available online at: https://www.qatarbiobank.org.qa/find-out-more#11 (Accessed 7 August, 2020)
27. Langhof, H., Kahrass, H., Sievers, S., et al. (2017). Access policies in biobank research: what criteria do they include and how publicly available are they? A cross-sectional study. European journal of human genetics : EJHG, 25(3), 293–300. https://doi.org/10.1038/ejhg.2016.172
28. OECD Guidelines for Human Biobanks and Genetic Research Databases (HBGRDs). Available online at : http://www.oecd.org/sti/emerging-tech/guidelines-for-human-biobanks-and-genetic-research-databases.htm. (Accessed 7 August 2020)
29. Knoppers B. M. (2014). Framework for responsible sharing of genomic and health-related data. The HUGO journal, 8(1), 3. https://doi.org/10.1186/s11568-014-0003
30. The proposed law of clinical medical research. Available at https://bit.ly/2EB6va8 (Accessed 7 August 2020).

31. The president objects to the law of clinical medical research. Available at https://www.almasryalyoum.com/news/details/1328846. (Accessed 7 August 2020).
32. Nicol D, Critchley C, McWhirter R, et al. (2016)Understanding public reactions to commercialization of biobanks and use of biobank resources. Soc Sci Med.;162:79–87. https://doi.org/10.1016/j.socscimed.2016.06.028
33. Alahmad G, Hifnawy T, Abbasi B, et al. Attitudes toward medical and genetic confidentiality in the Saudi research biobank: An exploratory survey. Int J Med Inform. 2016;87:84-90. doi:10.1016/j.ijmedinf.2015.12.015
34. Matandika, L., Ngóngóla, R. T., Mita, K., et al. (2020). A qualitative study exploring stakeholder perspectives on the use of biological samples for future unspecified research in Malawi. BMC medical ethics, 21(1), 61. https://doi.org/10.1186/s12910-020-00503-4
35. Egypttoday. Sisi endorses law on personal data protection. Available at: https://www.egypttoday.com/Article/1/89794/Sisi-endorses-law-onpersonal-data-protection. (Accessed 7 August 2020).
36. Cadigan, R., Easter, M., Dobson, A. et al. (2011)“That's a good question”: University researchers' views on ownership and retention of human genetic specimens. GenetMed 13, 569–575 https://doi.org/10.1097/GIM.0b013e318211a9c2
37. Yassin, R., Lockhart, N., González del Riego, M., et al.. (2010). Custodianship as an ethical framework for biospecimen-based research. Cancer epidemiology, biomarkers & prevention : a publication of the American Association for Cancer Research, cosponsored by the American Society of Preventive Oncology, 19(4), 1012–1015. https://doi.org/10.1158/1055-9965.EPI-10-0029

38. Fullerton SM, Anderson NR, Guzauskas G, Freeman D, Fryer-Edwards K. Meeting the governance challenges of next-generation biorepository research. Sci Transl Med. 2010;2(15):15cm3. doi:10.1126/scitranslmed.3000361
39. Matzke, L. A., Babinszky, S., Slotty, A., Meredith, A., Castillo-Pelayo, T., Henderson, M. K., Simeon-Dubach, D., Schacter, B., & Watson, P. H. (2017). Biospecimen User Fees: Global Feedback on a Calculator Tool. Biopreservation and biobanking, 15(1), 57–64. https://doi.org/10.1089/bio.2016.0027
40. Common Minimum Technical Standards and Protocols for Biobanks Dedicated to Cancer Research (2017). Available online at : https://www.iarc.fr/media-centre-iarc-news-technicalreport-44/ (Accessed 7 August 2020).
41. Strech, D., Bein, S., Brumhard, M., et al. (2016). A template for broad consent in biobank research. Results and explanation of an evidence and consensus-based development process. European Journal of Medical Genetics, 59(6-7), 295–309. doi:10.1016/j.ejmg.2016.04.002.
42. Hansson, M. Ethics and biobanks. Br J Cancer 100, 8–12 (2009). https://doi.org/10.1038/sj.bjc.6604795.
43. Mikkelsen, R. B., Gjerris, M., Waldemar, G., et al. (2019). Broad consent for biobanks is best - provided it is also deep. BMC medical ethics, 20(1), 71. https://doi.org/10.1186/s12910-019-0414-6
44. Ethics and Governance Framework for Best Practice in Genomic Research and Biobanking in Africa. (2017). Available online at https://www.sun.ac.za/english/faculty/healthsciences/rdsd/Documents/Final%20Framework%20for%20African%20genomics%20and%20biobanking_SC_February%202017%20II%20.pdf (Accessed 7 August 2020)

45. NCI Best Practices for Biospecimen Resources (2016). Available online at https://biospecimens.cancer.gov/bestpractices/2016-NCIBestPractices.pdf (Accessed 7 August 2020)
46. ISBER Best Practices 4th Edition (2018). Available online at https://www.isber.org/page/BPDownload4ed (Accessed 7 August 2020).
47. Alahmad G, Alzahrany H, Almutairi AF. Returning Results of Stored Biological Samples and Biobanks: Perspectives of Saudi Arabian Biomedical Researchers [published online ahead of print, 2020 Jul 20]. Biopreserv Biobank. 2020;10.1089/bio.2020.0002. doi:10.1089/bio.2020.0002.
48. Alahmad, G., & Dierickx, K. (2017). Return of Research Results in the Saudi Biobank: An Exploratory Survey. Genetic Testing and Molecular Biomarkers, 21(3), 166–170. doi:10.1089/gtmb.2016.0396
49. Bledsoe, M. J., Grizzle, W. E., Clark, B. J., et al. (2012). Practical implementation issues and challenges for biobanks in the return of individual research results. Genetics in medicine : official journal of the American College of Medical Genetics, 14(4), 478–483. https://doi.org/10.1038/gim.2011.67

Click here to access/download
Supporting Information
Supplementary files.docx
