# Supplementary material for: Knowledge, perceptions and attitude of Egyptian physicians towards biobanking issues
Source: PLoS One. 2021 Mar 26;16(3):e0248401. doi: 10.1371/journal.pone.0248401 (PMC7996976; doi:10.1371/journal.pone.0248401)
Supplement: Attachment — Submitted filename: PONE_D_34081 Review Comments.pdf [file pone.0248401.s006.pdf]

Abstract.

Page 8 –

Methods section: Authors should add more information on the coverage of the survey. Was a nationally representative database of physicians used, and so the survey was nationally representative?

The phrase "and communicated with the group..." can be revised for better clarity. What was communicated to the target group?

Introduction

General note:
The authors have written this section in the simplest form, which generally is good, capturing highlights about biobanking. However, I find a little bit of problem with the synchrony of the paragraphs leading to the justification of why this paper was written. Especially, the link between why stakeholder engagement is needed and thus, this study on stakeholder perception is not fluid enough to support a convincing justification.

I seem to think that paragraphs 3 ("One of the biggest….") and 4 ("Stakeholders can…") can be modified to make the argument on why stakeholder engagement is needed to support a governance and regulation regime for biobanks in Egypt, which would be expected to further support ethical biobanking research in the country. This new paragraph can then come after the submission about lack of regulations and linked with the sentence that reads: "The development of local ethics guidelines…".

Page 10
Paragraph 2, first sentence – "Both genomic research population biobanks are growing….". There is literature on types of biobanks, which the authors should cite. I have not come across a scholarly reference that has listed "genomic research" as a type of biobank. I am aware of the following types: population-based, disease-specific, tissue, DNA/RNA (or more broadly, "genetic material" and virtual biobanks. All these can support genomics research. A citation to support the biobank types listed by the authors should help. Adjustments may be necessary for the absence of relevant citation to conform with what is more widely documented.

Alternatively, the authors may wish to modify the opening sentence of this paragraph. Something like: "Genomic research supported by population biobanks is growing worldwide". Just a suggestion of how the submission could be better clarified.

Page 11
The first sentence on the page. Consider changing the words "communication" and "engagement" to their present participle forms for tense agreement with the lead word, "identifying".

Last paragraph before the section on "Subjects and methods" – The sentence that reads: "In our previous work….", leaves the reader wondering which issues were studied. I would suggest the authors should review this sentence to indicate that in previous work, they reported on knowledge and attitude of patients towards biobanking in Egypt. That building on

the report from patients, in this paper, they are reporting on what physicians know and their attitudes towards biobanking.

Page 12

Study design, Participants, Sampling and Setting
The third sentence that reads “the physicians were recruited…”, can be more clearly written. I would like to see recruitment and questionnaire response as two separate activities. My understanding is that participants were invited to participate in the study via e-mails and through social media. The questionnaire was designed on Google Forms and those that agreed to participate were asked to use the link to the questionnaire provided initially to respond to the survey. Perhaps this much explanation can be moved to the section on “Study too and data collection”, and simply delete the sentence “the physicians were recruited” from this section.

Study tool and data collection
The second sentence, “study started with an introduction explaining what biobanks are…” is lacking essential details to understand what was done. By saying “the survey started…”, are the authors saying that this bit was contained in the questionnaire, or was it preliminary sort of recruitment information provided? This clarification needs to be made and necessary adjustments to the way it is reported here also made. If it was the former, it would make more meaning to simply highlight the different sections of the questionnaire. If it was the latter, the authors should be clear about that and also indicate how this information was provided to persons recruited via email vs those via social media.

Here’s what I am guessing happened. A text containing background information on biobanks and links to other biobank information resources and another to the survey questionnaire was sent either to emails of identified potential participants or a social media handle dedicated for this study. Upon reading the informational text, individuals willing to participate were invited to open the questionnaire link to respond to the survey. The survey questionnaire had mainly three sections – a) demographic information; b) basic knowledge about biobanking before the survey; and c) perceptions and attitudes on biobanking.

However, these are my thoughts which I thought I should share so the authors could see why I feel the section, if meaning to convey what my thoughts are, needs to be modified to reflect same. Clarity and synchrony in the conveyance of the information intended are key.

Overall, I would like to invite the authors to further review this section for improved clarity and brevity.

Page 13
Ethical considerations
I do think that the statement provided, which was intended to facilitate an opt-out consent model was inadequate in itself to be accepted and referred to as ‘informed consent’. I would like to understand if the statement referred to by the authors (“By completing this questionnaire…”), was the only information provided to the participants to obtain their consent. Authors should consider writing out summarily how consent was obtained if more than this statement was used in the process.

Authors should be more specific highlights on mechanisms adopted to keep data confidential.

Results

Page 8, last sentence. It is not clear why the authors chose to classify the participant responses following the last sentence (“Moreover, the following statements…”) as indicating uncertainty among the physicians. Rather I would say the statements show issues for which the physicians were less in favour of regarding biobanking.

Page 10, paragraph 1. While indeed the sentences and structure in which the results were presented here could be understood, with much attention, I think there is room for improved clarity. I also do not think it was appropriate to group disagreement and uncertainty in the responses. I would suggest that these should be separated since already ‘disagreement’ covered “strongly disagree” and “disagree. If this is done, you would see that except for items 3 and 7, most participants agreed with the statements proffered.

**Total Disagree (strongly disagree and disagree)**	**Total Agree (strongly agree and agree)**
9.4	73.1
30.5	42.6
38.1	31.9
4.9	82.1
27.3	44.8
22.5	56.1
41.3	20.2

Page 17
On the level of agreement on the governance issues: I would suggest we also add the statements that responds least agreed with – “Biobanks own the stored samples (35.4%)”.

On the level of agreement of the respondents to survey items regarding consent, authors should also consider at least reporting the level of agreement on broad consent (47.9%) and expectations to receive some of form of compensation (48.5%)

Page 18
On the last sentence on associations between demographic and biobanking KAP variables, authors should consider changing “…and the type of research of current or future research of the participant” to “…and the current or future research interests of the participant”.

Page 19
Knowledge about biobanking – authors should provide the actual proportion of participants that have prior knowledge about biobanks. The first sentence should also be corrected to indicate specifically, the variable that the authors are talking about here – is it knowledge about biobanks in general or that of the existence of biobanks?

With a careful review, this section can be markedly improved brevity and clarity-wise. For example, the second sentence here can be modified to read: “In a similar survey among patients, we reported that more than 80% of participants had never heard about the term, “biobanking”, before the survey.

The use of the word “that’s” in the tenth sentence is rather informal and should be corrected, please. Authors should also be diligent in making these sorts of corrections in relevant parts of the manuscript.

Page 20

General attitude towards biobanking – Authors could modify the sixth sentence to start with “For example, a study similar that being reported here showed a negative correlation…..”.

Page 21
“Perceptions and attitude…. and sharing of samples” –

Provide relevant reference(s) for the sentence on sharing samples with commercial entities - “Sharing of samples across borders and with commercial….”

Page 27

Limitations
I think the limitations reported are valid, but authors should provide an additional a phrase or a sentence to indicate why the two issues mentioned were limitations. In addition, I would like to encourage the authors to think of other important limitations that they may have missed. For example, was the online-based survey the best approach to optimally reach this sample population? Are there already biases from the approach for this study towards professionals that are social media savvy, and how might that affect representativeness?
